# Co^II^-catalysed synthesis of *N*-(4-meth­oxy­phen­yl)-5-(pyridin-4-yl)-1,3,4-oxa­diazol-2-amine hemi­hydro­chloride monohydrate

**DOI:** 10.1107/S2056989024002044

**Published:** 2024-03-12

**Authors:** Ram N Gautam, Sankatha P Sonkar, Shailendra Yadav, Paras Nath, Manoj K. Bharty

**Affiliations:** a Department of Chemistry, Banaras Hindu University, Varanasi-221005, India; b Department of Chemistry, AKS University, Satna-485001, India; c Buddha PG College, Kushinagar - 274403, India; University of Aberdeen, United Kingdom

**Keywords:** 1,3,4-oxa­diazole, *cyclo*-desulfurization, Hirshfeld surface analysis, crystal structure

## Abstract

The title compound, C_14_H_12_N_4_O_2_·0.5HCl·H_2_O or H(C_14_H_12_N_4_O_2_)_2_^+^·Cl^−^·2H_2_O, arose from the unexpected cyclization of isonicotinoyl-*N*-phenyl hydrazine carbo­thio­amide catalysed by cobalt(II) acetate. The organic mol­ecule is almost planar and a symmetric N⋯H^+^⋯N hydrogen bond links two of them together, with the H atom lying on a crystallographic twofold axis. The extended structure features N—H⋯O and O—H⋯Cl hydrogen bonds, which generate [001] chains. Weak C—H⋯Cl inter­actions cross-link the chains. The chloride ion has site symmetry 2. The major contributions to the Hirshfeld surface are from H⋯H (47.1%), Cl⋯H/H⋯Cl (total 10.8%), O⋯H/H⋯O (7.4%) and N⋯H/H⋯N (6.7%) inter­actions.

## Abstract 2

The Co^II^-catalysed synthesis and crystal structure is reported for the title compound, which features a symmetric N⋯H^+^⋯N unit.

## 1. Chemical context

1,3,4-Oxa­diazole derivatives have been studied in recent years for their diverse biological activities (Gond *et al.*, 2023 ▸; Abd-Ellah *et al.*, 2017 ▸; Bitla *et al.*, 2020 ▸). As a result of their electron-accepting properties, high quantum yield, and good thermal and chemical stabilities, they have also been used in electroluminescent, optical and electron-transporting materials and chelating agents (Najare *et al.*, 2020 ▸; Wu *et al.*, 2012 ▸). Several methods for the synthesis of 1,3,4-oxa­diazo­les from acyclic precursors are available, which include oxidative cyclization of acyl­hydrazones (Jedlovská & Leško, 1994 ▸) and acyl­thio­semicarbazides (Omar *et al.*, 1996 ▸, Paswan *et al.*, 2015 ▸). In the presence of a strong acid, an *N*-acyl­hydrazine carbodi­thio­ate is converted into a thia­diazole whereas in the presence of a weak acid or base or on complexation they can be cyclized into oxa­diazole (Reid & Heindel, 1976 ▸; Jasinski *et al.*, 2011 ▸). 
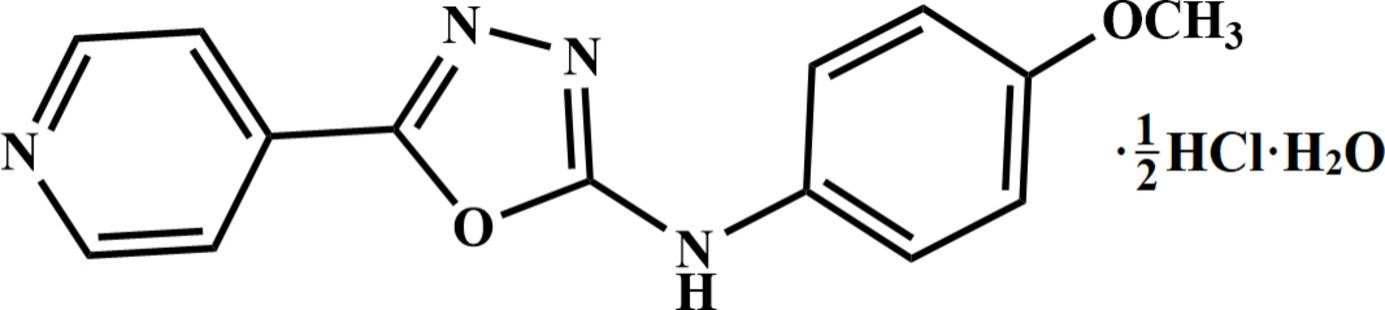


We have previously reported the cyclo-desulfurization of several *N*-acyl­hydrazine carbodi­thio­ates into the corres­ponding 1,3,4-oxa­diazole in the presence of manganese(II) acetate *via* the loss of H_2_S where the Mn^II^ ion presumably behaves as a weak Lewis acid (Paswan *et al.*, 2015 ▸, 2016 ▸; Gond *et al.*, 2022 ▸). In the present work, a similar reaction is reported in presence of Co^II^ chloride. Similar Co^II^-assisted cyclization reactions are also reported in the literature (Li *et al.*, 2021 ▸, 2023 ▸; Bharty *et al.*, 2012 ▸).

## 2. Structural commentary

**Figure 1:**
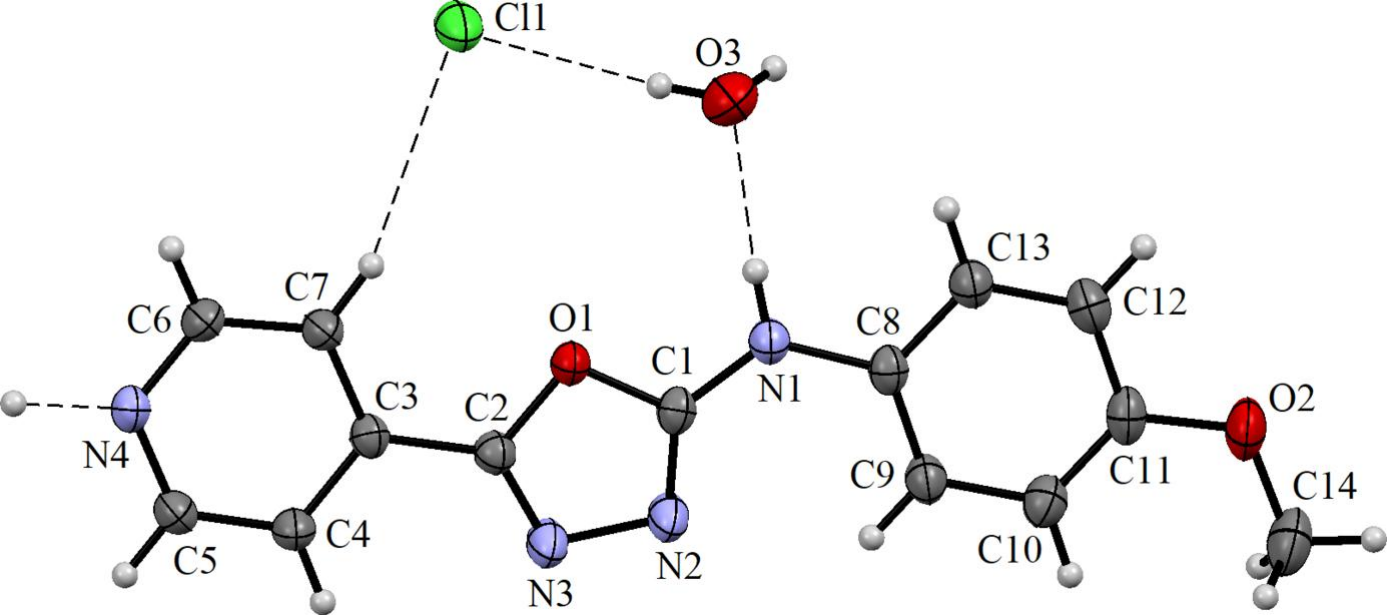
The mol­ecular structure of the title compound showing 30% probability displacement ellipsoids with hydrogen bonds indicated by dashed lines.

**Figure 2:**
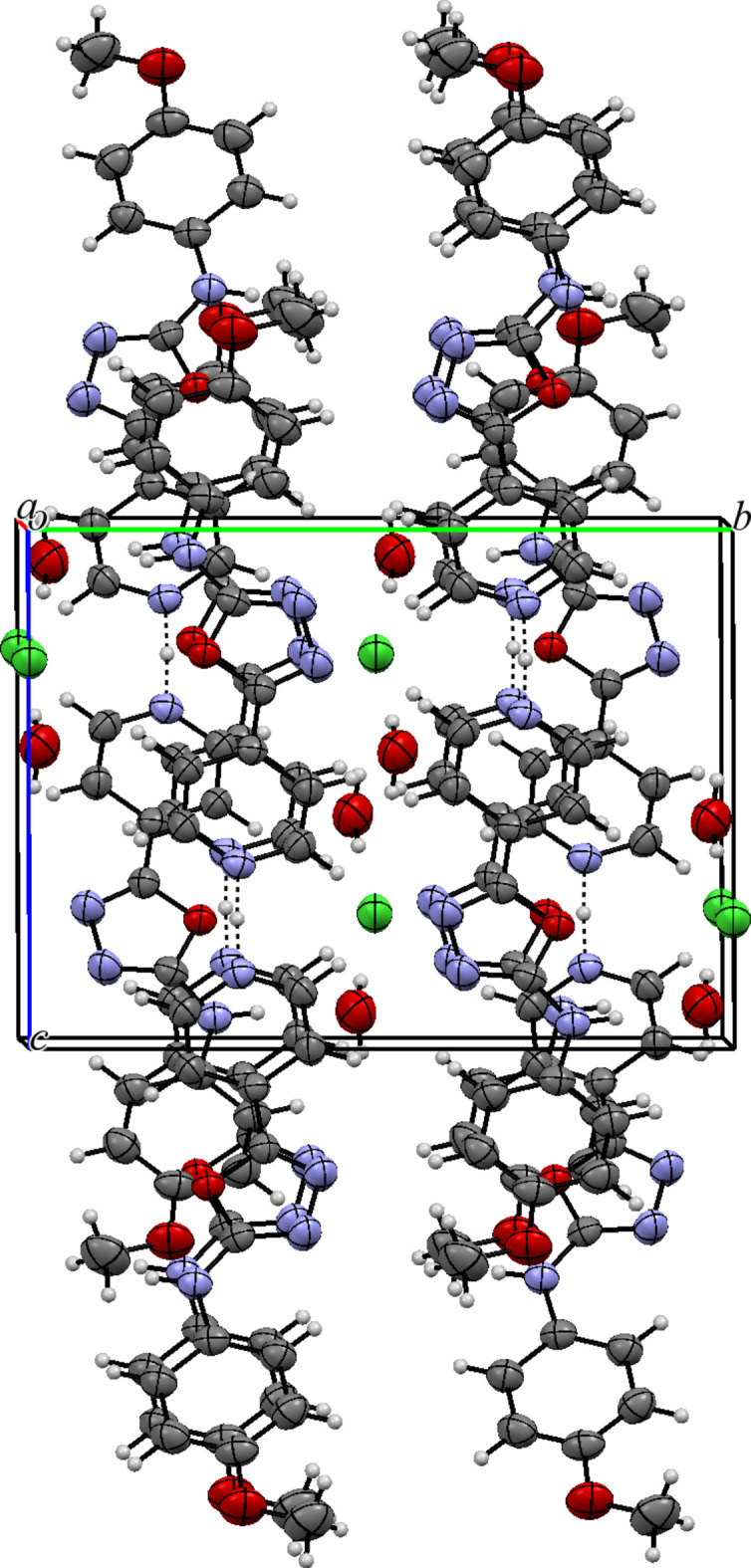
The packing of the title compound viewed along the *a*-axis direction.

The compound crystallizes in the monoclinic crystal system in space group *C*2/*c*. The asymmetric unit consists of one organic mol­ecule, half an equivalent of HCl and one water mol­ecule (Fig. 1 ▸). The C3–C7/N4 pyridyl, C1/C2/N2/N3/O1 oxa­diazole and C8–C13 phenyl rings are close to co-planar with the dihedral angles between pyridyl and oxa­diazole rings being 4.88 (9)°, oxa­diazole and phenyl rings 4.27 (10)° and pyridyl and phenyl rings 2.27 (9)°. The bond distances and angles of the 1,3,4-oxa­diazole ring [C1—N2 = 1.298 (2); C2—N3 = 1.277 (2) Å] are in good agreement with values reported previously (Jasinski *et al.*, 2011 ▸; Paswan *et al.*, 2015 ▸, 2016 ▸; Singh *et al.*, 2007 ▸). The C—N bond distance in the pyridine ring, C5—N4 = 1.336 (2) Å, is slightly longer than the corresponding bond in a similar compound (1.326 (2) Å; Singh *et al.*, 2006 ▸), probably due to the N⋯H inter­action (Fig. 2 ▸).

## 3. Supra­molecular features

**Figure 3:**
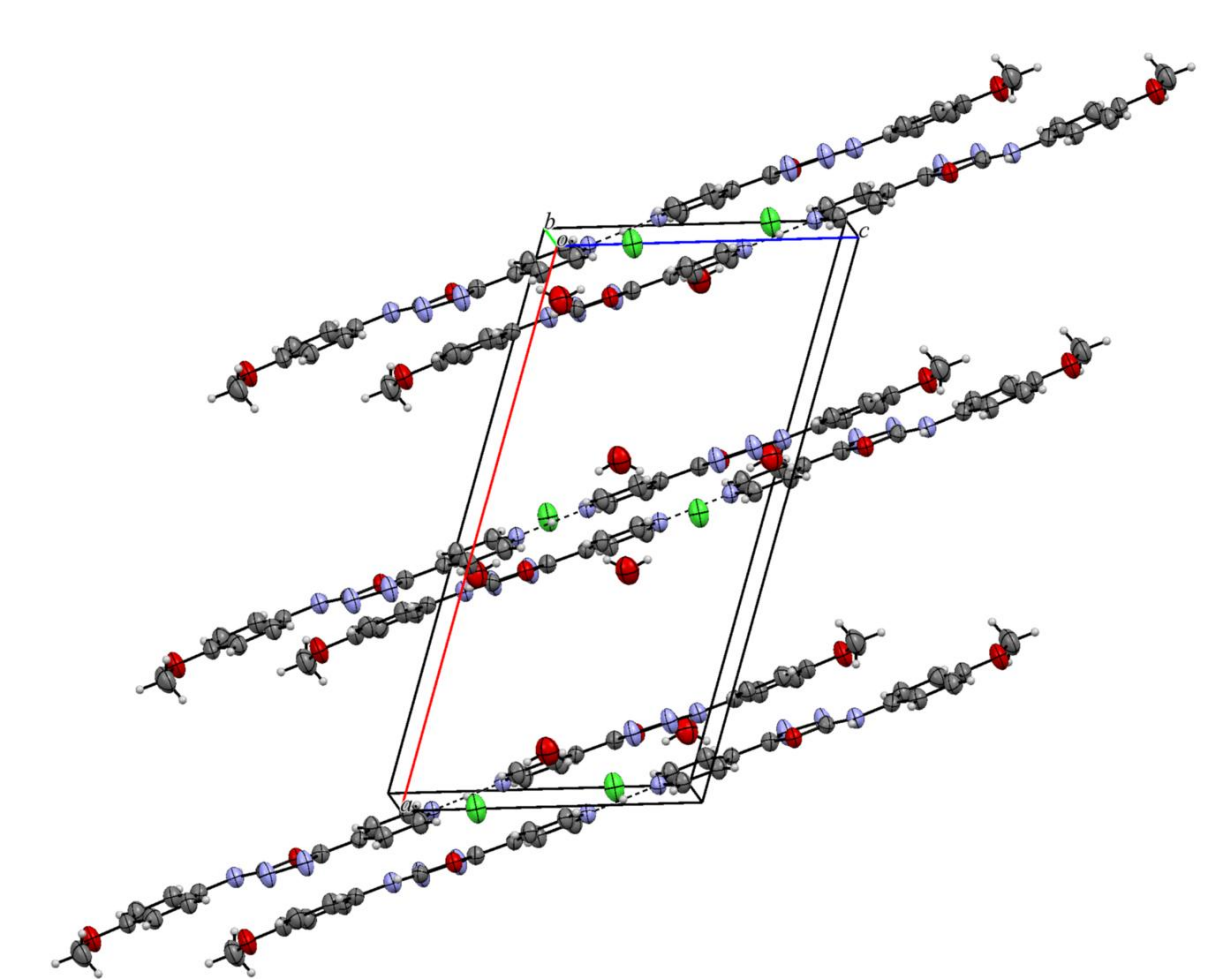
The packing of title compound viewed along the *b*-axis direction.

In the extended structure, two organic mol­ecules are linked through their pyridine nitro­gen atoms *via* the proton of the hydro­chloric acid, which lies on a crystallographic twofold axis. This strong, symmetrical, almost linear N4⋯H4*N*⋯N4 hydrogen bond (Table 1 ▸) leads to a rod-like dimeric structure. These units form a layer-like structure when viewed along *b* axis of the unit cell (Fig. 3 ▸). The water mol­ecules and chloride ions (site symmetry 2) are embedded in the space between the chains and are connected to them *via* N—H⋯O and O—H⋯Cl hydrogen bonds, thereby generating [001] chains. Weak C—H⋯Cl inter­actions are also observed (Table 1 ▸; Fig. 2 ▸).

## 4. Hirshfeld Surface Analysis

**Figure 4:**
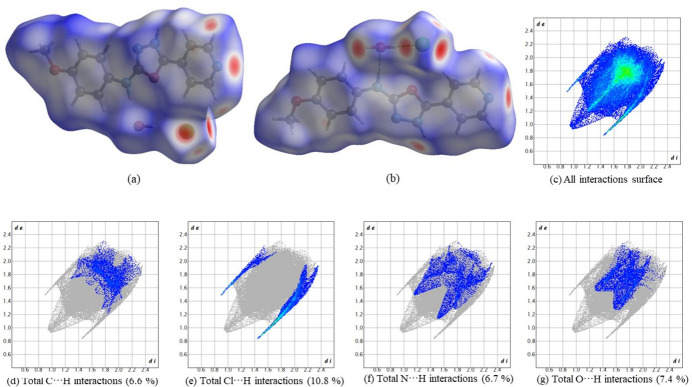
(*a*), (*b*) Two views of the Hirshfeld surface of the title compound mapped over *d_norm_*, (*c*) fingerprint plot showing the total contribution of individual inter­actions and those delineated into (*d*) C⋯H/H⋯C inter­actions (6.6%), (*e*) Cl⋯H/H⋯Cl inter­actions (10.8%), (*f*) N⋯H/H⋯N inter­actions (6.7%) and (*g*) O⋯H/H⋯O inter­actions (7.4%).

To gain further insight into the inter­molecular inter­actions, a Hirshfeld surface analysis was performed using *Crystal Explorer 17.5* (Spackman *et al.*, 2021 ▸). Fig. 4 ▸ *a*,*b* shows the Hirshfeld surface mapped over *d*_norm_. The red spots show the various hydrogen bonds noted above.

The two-dimensional fingerprint plots are presented in Fig. 4 ▸ *c*–*g*. The H⋯H (van der Waals) contacts dominate at 47.1%. Among the directional inter­actions present in the structure, the Cl⋯H/H⋯Cl (total 10.8%) contact is the most significant. Other contacts include 6.6% for C⋯H/H⋯C, 6.7% N⋯H/H⋯N and 7.4% for O⋯H/H⋯O inter­actions.

## 5. Synthesis and crystallization

**Figure 5:**
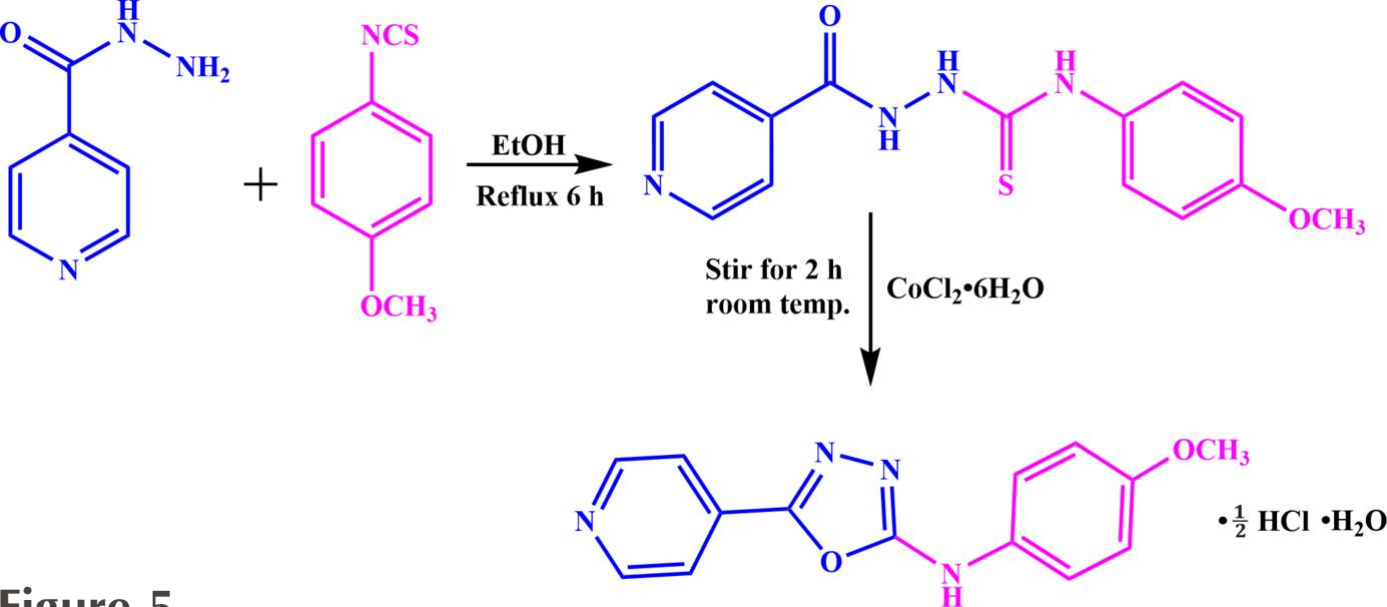
Synthesis scheme for the title compound.

2-Isonicotinoyl-*N*-(4-meth­oxy­phen­yl)hydrazine-1-carbo­thio­amide was prepared by adding 1.652 g (10.00 mmol) of 4-meth­oxy phenyl iso­thio­cyanate in ethanol solution to 1.370 g (10.00 mmol) of isonicotinohydrazide and the reaction mixture was refluxed for 6 h at 333 K. Upon cooling, a white precipitate of 2-isonicotinoyl-*N*-(4-meth­oxy­phen­yl)hydrazine-1-carbo­thio­amide was obtained (Fig. 5 ▸), which was filtered off and washed with a 50:50 *v*/*v* mixture of water and ether. Then, 1.00 mmol of 2-isonicotinoyl-*N*-(4-meth­oxy­phen­yl)hydrazine-1-carbo­thio­amide was dissolved in a 50:50 *v*/*v* mixture of methanol and chloro­form, and a methano­lic solution of 0.5 mmol of CoCl_2_·6H_2_O was added and stirred for 2 h, during which time the smell of H_2_S was noted. The clear solution obtained was kept for crystallization and after 15 days, pale-pink blocks of the title compound were grown. Yield: 60.6%; m.p. 495–498 K. Analysis calculated for C_14_H_12_N_4_O_2._0.5 HCl·H_2_O: C, 55.21; H, 4.79; N, 18.39%; found: C, 55.25; H, 4.50; N, 18.55%.

The IR spectrum (KBr disc) shows an absorption band at 3280 cm^−1^ due to the NH group. The C=O band is absent and a new band is observed at 1623 cm^−1^ corresponding to the C=N bond. In addition, a blue shift is observed for the N=N band at 1179 cm^−1^ compared to the single bond in the thio­semicarbazide inter­mediate (Fig. 1 in the supporting information). All these data indicate that the carbo­thio­amide moiety has been transformed into the corresponding oxa­diazole (Chandra *et al.*, 2022 ▸; Jaiswal *et al.*, 2023*a* ▸ *b* ▸).

The ^1^H NMR spectrum of the title compound in DMSO-*d6* displays peaks at *δ* 10.69 ppm due to the NH proton, at *δ* 8.91 and 7.90 ppm due to the pyridyl ring protons and at δ 7.55 and 6.98 ppm due to phenyl ring protons. The meth­oxy protons appear at δ 3.74 ppm. (Fig. 2 in the supporting information). In the ^13^C NMR spectrum, peaks at *δ* 156.9 and 155.2 ppm arise from oxa­diazole ring carbon atoms, the meth­oxy C atom appears at 55.7 ppm and the phenyl and pyridyl carbon atoms are observed in the range *δ* 114.8–132.4 ppm (Fig. 3 in the supporting information). An absorption at 338 nm in the electronic spectrum of the title compound can be attributed to its π–π* transition (Fig. 4 in the supporting information). It displays fluorescence at 418 nm upon excitation at 338 nm (Fig. 5 in the supporting information) when dissolved in 10^−5^ *M* DMSO solution.

## 6. Refinement

Crystal data, data collection and structure refinement details are summarized in Table 2 ▸. Atom H4*N* was freely refined. Other H atoms were placed in idealized locations (N—H = 0.86 Å, C—H = 0.93–0. 96 Å) and refined using a riding model with *U*_iso_(H) =1.2*U*_eq_(C,N) or 1.5*U*_eq_(C-meth­yl). Asymmetric N—H⋯N/N⋯H—N refinements with the H atom displaced towards one of the N atoms were inconclusive and atom H4*N* was placed on the twofold axis.

## Supplementary Material

Crystal structure: contains datablock(s) I. DOI: 10.1107/S2056989024002044/hb8088sup1.cif

Structure factors: contains datablock(s) I. DOI: 10.1107/S2056989024002044/hb8088Isup2.hkl

Spectra. DOI: 10.1107/S2056989024002044/hb8088sup3.docx

Supporting information file. DOI: 10.1107/S2056989024002044/hb8088Isup4.cml

CCDC reference: 2238764

Additional supporting information:  crystallographic information; 3D view; checkCIF report

## Figures and Tables

**Table 1:** Hydrogen-bond geometry (Å, °)

*D*—H⋯*A*	*D*—H	H⋯*A*	*D*⋯*A*	*D*—H⋯*A*
N4—H4*N*⋯N4^i^	1.34 (1)	1.34 (1)	2.675 (3)	178 (3)
N1—H1⋯O3	0.86	2.02	2.875 (2)	172
O3—H22⋯Cl1	0.82 (4)	2.43 (4)	3.233 (2)	166 (3)
O3—H21⋯Cl1^ii^	0.81 (4)	2.55 (4)	3.359 (3)	172 (4)
C7—H7⋯Cl1	0.93	2.93	3.7940 (18)	155
C6—H6⋯Cl1^iii^	0.93	2.82	3.7137 (18)	163
C5—H5⋯N3^iv^	0.93	2.56	3.329 (2)	140
C9—H9⋯N2	0.93	2.34	2.969 (2)	125

Symmetry codes: (i) 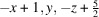 ; (ii) 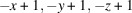 ; (iii) 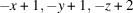 ; (iv) 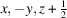 .

**Table 2:** Experimental details

Crystal data	
Chemical formula	C_28_H_25_N_8_O_4_^+^·Cl^−^·2H_2_O
*M*_r_	609.04
Crystal system, space group	Monoclinic, *C*2/*c*
Temperature (K)	293
*a*, *b*, *c* (Å)	20.5406 (9), 13.7457 (5), 10.5650 (3)
β (°)	107.006 (4)
*V* (Å^3^)	2852.54 (19)
*Z*	4
Radiation type	Mo *K*α
μ (mm^−1^)	0.19
Crystal size (mm)	0.32 × 0.26 × 0.24

Data collection	
Diffractometer	Bruker multiwire proportional
No. of measured, independent and observed [*I* > 2σ(*I*)] reflections	16772, 3121, 2026
*R*_int_	0.029
(sin θ/λ)_max_ (Å^−1^)	0.639

Refinement	
*R*[*F* ^2^ > 2σ(*F* ^2^)], *wR*(*F* ^2^), *S*	0.041, 0.119, 0.98
No. of reflections	3009
No. of parameters	205
H-atom treatment	H atoms treated by a mixture of independent and constrained refinement
Δρ_max_, Δρ_min_ (e Å^−3^)	0.20, −0.18

Computer programs: FRAMBO (Bruker, 2004 ▸), *SHELXTL* (Sheldrick, 2008 ▸) and *SHELXL2018/3* (Sheldrick, 2015 ▸).

## supplementary crystallographic information

### Crystal data

**Table:**

C_28_H_25_N_8_O_4_^+^·Cl^−^·2H_2_O	*F*(000) = 1276
*M**_r_* = 609.04	*D*_x_ = 1.418 Mg m^−^^3^
Monoclinic, *C*2/*c*	Mo *K*α radiation, λ = 0.71073 Å
*a* = 20.5406 (9) Å	Cell parameters from 8519 reflections
*b* = 13.7457 (5) Å	θ = 2.5–26.2°
*c* = 10.5650 (3) Å	µ = 0.19 mm^−^^1^
β = 107.006 (4)°	*T* = 293 K
*V* = 2852.54 (19) Å^3^	Block, pinkish
*Z* = 4	0.32 × 0.26 × 0.24 mm

### Data collection

**Table:**

Bruker multiwire proportional diffractometer	*R*_int_ = 0.029
Radiation source: sealed tube	θ_max_ = 27.0°, θ_min_ = 2.5°
phi and ω scans	*h* = −25→25
16772 measured reflections	*k* = −17→13
3121 independent reflections	*l* = −13→11
2026 reflections with *I* > 2σ(*I*)	

### Refinement

**Table:**

Refinement on *F*^2^	Primary atom site location: dual
Least-squares matrix: full	Hydrogen site location: mixed
*R*[*F*^2^ > 2σ(*F*^2^)] = 0.041	H atoms treated by a mixture of independent and constrained refinement
*wR*(*F*^2^) = 0.119	*w* = 1/[σ^2^(*F*_o_^2^) + (0.0602*P*)^2^ + 1.1461*P*] where *P* = (*F*_o_^2^ + 2*F*_c_^2^)/3
*S* = 0.98	(Δ/σ)_max_ < 0.001
3009 reflections	Δρ_max_ = 0.20 e Å^−^^3^
205 parameters	Δρ_min_ = −0.18 e Å^−^^3^
0 restraints	

### Special details

**Table:**

Geometry. All esds (except the esd in the dihedral angle between two l.s. planes) are estimated using the full covariance matrix. The cell esds are taken into account individually in the estimation of esds in distances, angles and torsion angles; correlations between esds in cell parameters are only used when they are defined by crystal symmetry. An approximate (isotropic) treatment of cell esds is used for estimating esds involving l.s. planes.

### Fractional atomic coordinates and isotropic or equivalent isotropic displacement parameters (Å^2^)

**Table:**

	*x*	*y*	*z*	*U*_iso_*/*U*_eq_	
Cl1	0.500000	0.49848 (5)	0.750000	0.0696 (3)	
O1	0.60240 (6)	0.24882 (8)	0.73971 (10)	0.0438 (3)	
O2	0.73500 (8)	0.20297 (12)	0.12535 (14)	0.0738 (4)	
N4	0.52376 (7)	0.20300 (10)	1.14485 (13)	0.0447 (4)	
N1	0.63480 (7)	0.26868 (11)	0.55203 (13)	0.0470 (4)	
H1	0.627028	0.329212	0.562248	0.056*	
N2	0.62683 (8)	0.11322 (11)	0.64893 (14)	0.0567 (4)	
O3	0.59808 (11)	0.46965 (13)	0.5629 (3)	0.0817 (5)	
N3	0.60728 (9)	0.08972 (11)	0.76220 (15)	0.0579 (4)	
C3	0.57017 (8)	0.18290 (12)	0.92666 (15)	0.0395 (4)	
C1	0.62287 (8)	0.20733 (13)	0.64050 (15)	0.0423 (4)	
C2	0.59381 (8)	0.16960 (12)	0.81088 (16)	0.0421 (4)	
C7	0.55180 (9)	0.27308 (13)	0.96298 (16)	0.0465 (4)	
H7	0.554704	0.328207	0.913884	0.056*	
C8	0.65878 (8)	0.24517 (13)	0.44342 (16)	0.0438 (4)	
C6	0.52914 (9)	0.27987 (13)	1.07303 (16)	0.0481 (4)	
H6	0.517179	0.340647	1.097866	0.058*	
C4	0.56536 (9)	0.10281 (13)	1.00308 (17)	0.0489 (4)	
H4	0.577659	0.041269	0.981505	0.059*	
C5	0.54218 (9)	0.11591 (13)	1.11088 (17)	0.0504 (5)	
H5	0.539154	0.062199	1.162390	0.060*	
C13	0.66769 (10)	0.32122 (14)	0.36423 (17)	0.0526 (5)	
H13	0.656561	0.384241	0.382362	0.063*	
C11	0.70949 (9)	0.21120 (15)	0.23119 (17)	0.0537 (5)	
C9	0.67449 (9)	0.15188 (14)	0.41413 (17)	0.0514 (5)	
H9	0.668165	0.100040	0.465852	0.062*	
C12	0.69280 (10)	0.30451 (15)	0.25909 (19)	0.0578 (5)	
H12	0.698590	0.356168	0.206558	0.069*	
C10	0.69972 (10)	0.13515 (15)	0.30768 (18)	0.0560 (5)	
H10	0.710046	0.072074	0.288146	0.067*	
C14	0.75778 (13)	0.11035 (19)	0.0984 (2)	0.0827 (7)	
H14A	0.774283	0.114498	0.022456	0.124*	
H14B	0.793818	0.088726	0.173472	0.124*	
H14C	0.720706	0.064931	0.081080	0.124*	
H22	0.5797 (18)	0.477 (2)	0.621 (3)	0.129 (14)*	
H21	0.578 (2)	0.478 (3)	0.485 (4)	0.159 (18)*	
H4N	0.500000	0.205 (2)	1.250000	0.092 (10)*	

### Atomic displacement parameters (Å^2^)

**Table:**

	*U*^11^	*U*^22^	*U*^33^	*U*^12^	*U*^13^	*U*^23^
Cl1	0.1079 (7)	0.0494 (4)	0.0619 (4)	0.000	0.0413 (4)	0.000
O1	0.0531 (7)	0.0434 (7)	0.0391 (6)	0.0010 (5)	0.0201 (5)	−0.0004 (5)
O2	0.0879 (11)	0.0901 (11)	0.0591 (8)	0.0004 (9)	0.0458 (8)	−0.0021 (8)
N4	0.0507 (9)	0.0484 (9)	0.0375 (7)	0.0013 (7)	0.0168 (6)	−0.0010 (6)
N1	0.0547 (9)	0.0476 (8)	0.0434 (8)	−0.0006 (7)	0.0218 (7)	0.0003 (7)
N2	0.0838 (12)	0.0460 (10)	0.0518 (9)	−0.0039 (8)	0.0380 (8)	−0.0053 (7)
O3	0.1057 (15)	0.0670 (11)	0.0814 (13)	0.0140 (9)	0.0413 (13)	−0.0026 (9)
N3	0.0875 (12)	0.0457 (9)	0.0520 (9)	−0.0028 (8)	0.0386 (9)	−0.0040 (7)
C3	0.0413 (9)	0.0428 (9)	0.0346 (8)	−0.0022 (7)	0.0115 (7)	−0.0011 (7)
C1	0.0421 (9)	0.0488 (10)	0.0371 (9)	−0.0036 (8)	0.0133 (7)	−0.0054 (7)
C2	0.0471 (10)	0.0406 (9)	0.0395 (9)	−0.0022 (7)	0.0141 (8)	−0.0009 (7)
C7	0.0604 (11)	0.0395 (9)	0.0421 (9)	0.0029 (8)	0.0189 (8)	0.0033 (7)
C8	0.0408 (9)	0.0546 (11)	0.0361 (8)	−0.0040 (8)	0.0116 (7)	−0.0016 (8)
C6	0.0597 (11)	0.0424 (10)	0.0457 (10)	0.0045 (8)	0.0209 (9)	−0.0018 (8)
C4	0.0628 (12)	0.0398 (10)	0.0493 (10)	0.0033 (8)	0.0245 (9)	0.0018 (8)
C5	0.0633 (12)	0.0445 (10)	0.0476 (10)	0.0016 (9)	0.0228 (9)	0.0079 (8)
C13	0.0592 (12)	0.0552 (11)	0.0469 (10)	−0.0007 (9)	0.0208 (9)	0.0019 (8)
C11	0.0504 (11)	0.0751 (14)	0.0397 (9)	−0.0067 (9)	0.0195 (8)	−0.0036 (9)
C9	0.0597 (12)	0.0548 (12)	0.0446 (10)	−0.0049 (9)	0.0231 (9)	0.0002 (8)
C12	0.0639 (13)	0.0655 (13)	0.0484 (10)	−0.0049 (10)	0.0235 (9)	0.0070 (9)
C10	0.0595 (12)	0.0595 (12)	0.0534 (11)	−0.0021 (9)	0.0236 (9)	−0.0085 (9)
C14	0.0935 (18)	0.0996 (19)	0.0706 (14)	−0.0045 (15)	0.0485 (13)	−0.0172 (13)

### Geometric parameters (Å, º)

**Table:**

O1—C1	1.3634 (19)	C7—H7	0.9300
O1—C2	1.3637 (19)	C8—C9	1.379 (3)
O2—C11	1.371 (2)	C8—C13	1.384 (2)
O2—C14	1.414 (3)	C6—H6	0.9300
N4—C6	1.324 (2)	C4—C5	1.369 (2)
N4—C5	1.336 (2)	C4—H4	0.9300
N4—H4N	1.3379 (15)	C5—H5	0.9300
N1—C1	1.334 (2)	C13—C12	1.374 (3)
N1—C8	1.412 (2)	C13—H13	0.9300
N1—H1	0.8600	C11—C10	1.371 (3)
N2—C1	1.298 (2)	C11—C12	1.382 (3)
N2—N3	1.407 (2)	C9—C10	1.388 (2)
O3—H22	0.82 (4)	C9—H9	0.9300
O3—H21	0.81 (4)	C12—H12	0.9300
N3—C2	1.277 (2)	C10—H10	0.9300
C3—C7	1.382 (2)	C14—H14A	0.9600
C3—C4	1.386 (2)	C14—H14B	0.9600
C3—C2	1.454 (2)	C14—H14C	0.9600
C7—C6	1.376 (2)		
			
C1—O1—C2	102.04 (13)	C7—C6—H6	118.8
C11—O2—C14	117.79 (17)	C5—C4—C3	118.79 (16)
C6—N4—C5	118.91 (15)	C5—C4—H4	120.6
C6—N4—H4N	124.9 (13)	C3—C4—H4	120.6
C5—N4—H4N	116.2 (13)	N4—C5—C4	122.43 (16)
C1—N1—C8	127.16 (15)	N4—C5—H5	118.8
C1—N1—H1	116.4	C4—C5—H5	118.8
C8—N1—H1	116.4	C12—C13—C8	120.64 (18)
C1—N2—N3	105.04 (14)	C12—C13—H13	119.7
H22—O3—H21	122 (4)	C8—C13—H13	119.7
C2—N3—N2	107.14 (14)	C10—C11—O2	125.05 (19)
C7—C3—C4	118.62 (16)	C10—C11—C12	119.59 (17)
C7—C3—C2	122.13 (15)	O2—C11—C12	115.36 (18)
C4—C3—C2	119.25 (15)	C8—C9—C10	120.20 (18)
N2—C1—N1	131.13 (16)	C8—C9—H9	119.9
N2—C1—O1	113.05 (14)	C10—C9—H9	119.9
N1—C1—O1	115.82 (15)	C13—C12—C11	120.19 (18)
N3—C2—O1	112.73 (15)	C13—C12—H12	119.9
N3—C2—C3	127.75 (15)	C11—C12—H12	119.9
O1—C2—C3	119.50 (14)	C11—C10—C9	120.31 (19)
C6—C7—C3	118.86 (16)	C11—C10—H10	119.8
C6—C7—H7	120.6	C9—C10—H10	119.8
C3—C7—H7	120.6	O2—C14—H14A	109.5
C9—C8—C13	119.05 (17)	O2—C14—H14B	109.5
C9—C8—N1	123.71 (16)	H14A—C14—H14B	109.5
C13—C8—N1	117.23 (16)	O2—C14—H14C	109.5
N4—C6—C7	122.38 (16)	H14A—C14—H14C	109.5
N4—C6—H6	118.8	H14B—C14—H14C	109.5
			
C1—N2—N3—C2	−0.2 (2)	C5—N4—C6—C7	1.3 (3)
N3—N2—C1—N1	−179.21 (18)	C3—C7—C6—N4	−0.6 (3)
N3—N2—C1—O1	0.22 (19)	C7—C3—C4—C5	0.5 (3)
C8—N1—C1—N2	−3.2 (3)	C2—C3—C4—C5	−179.14 (16)
C8—N1—C1—O1	177.42 (14)	C6—N4—C5—C4	−1.2 (3)
C2—O1—C1—N2	−0.20 (18)	C3—C4—C5—N4	0.3 (3)
C2—O1—C1—N1	179.33 (14)	C9—C8—C13—C12	−1.0 (3)
N2—N3—C2—O1	0.0 (2)	N1—C8—C13—C12	178.02 (16)
N2—N3—C2—C3	178.69 (16)	C14—O2—C11—C10	−5.0 (3)
C1—O1—C2—N3	0.08 (18)	C14—O2—C11—C12	175.28 (18)
C1—O1—C2—C3	−178.69 (14)	C13—C8—C9—C10	0.9 (3)
C7—C3—C2—N3	−174.38 (18)	N1—C8—C9—C10	−178.10 (15)
C4—C3—C2—N3	5.2 (3)	C8—C13—C12—C11	0.0 (3)
C7—C3—C2—O1	4.2 (2)	C10—C11—C12—C13	1.1 (3)
C4—C3—C2—O1	−176.23 (15)	O2—C11—C12—C13	−179.10 (17)
C4—C3—C7—C6	−0.3 (3)	O2—C11—C10—C9	178.98 (18)
C2—C3—C7—C6	179.26 (15)	C12—C11—C10—C9	−1.3 (3)
C1—N1—C8—C9	−0.8 (3)	C8—C9—C10—C11	0.3 (3)
C1—N1—C8—C13	−179.82 (16)		

### Hydrogen-bond geometry (Å, º)

**Table:**

*D*—H···*A*	*D*—H	H···*A*	*D*···*A*	*D*—H···*A*
N4—H4*N*···N4^i^	1.34 (1)	1.34 (1)	2.675 (3)	178 (3)
N1—H1···O3	0.86	2.02	2.875 (2)	172
O3—H22···Cl1	0.82 (4)	2.43 (4)	3.233 (2)	166 (3)
O3—H21···Cl1^ii^	0.81 (4)	2.55 (4)	3.359 (3)	172 (4)
C7—H7···Cl1	0.93	2.93	3.7940 (18)	155
C6—H6···Cl1^iii^	0.93	2.82	3.7137 (18)	163
C5—H5···N3^iv^	0.93	2.56	3.329 (2)	140
C9—H9···N2	0.93	2.34	2.969 (2)	125

Symmetry codes: (i) −*x*+1, *y*, −*z*+5/2; (ii) −*x*+1, −*y*+1, −*z*+1; (iii) −*x*+1, −*y*+1, −*z*+2; (iv) *x*, −*y*, *z*+1/2.
