# Supplementary material for: CoII-catalysed synthesis of N-(4-meth­oxy­phen­yl)-5-(pyridin-4-yl)-1,3,4-oxa­diazol-2-amine hemi­hydro­chloride monohydrate
Source: Acta Crystallogr E Crystallogr Commun. 2024 Mar 12;80(Pt 4):351–4. doi: 10.1107/S2056989024002044 (PMC10993596; doi:10.1107/S2056989024002044)
Supplement: Supplementary file 3 [file e-80-00351-sup3.docx]

**Co(II) catalyzed synthesis of N-(4-methoxyphenyl)-5-(pyridin-4-yl)-1,3,4-oxadiazol-2-amine hemihydrochloride monohydrate**

Ram N Gautam, Sankatha P Sonkar, Shailendra Yadav, Paras Nath and Manoj K Bharty*

**Supplementary Figures**

**
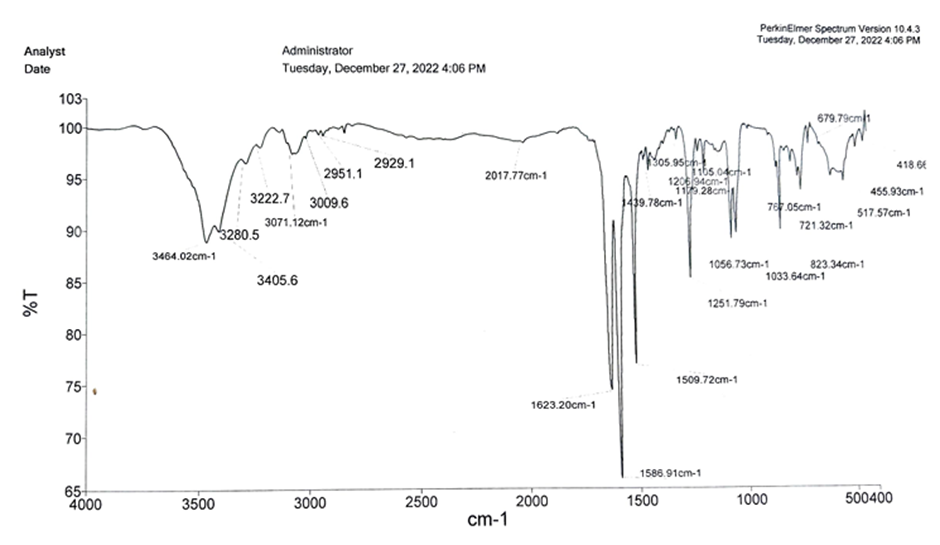
**

**Suppl. Fig. 1.** IR spectrum of title compound.


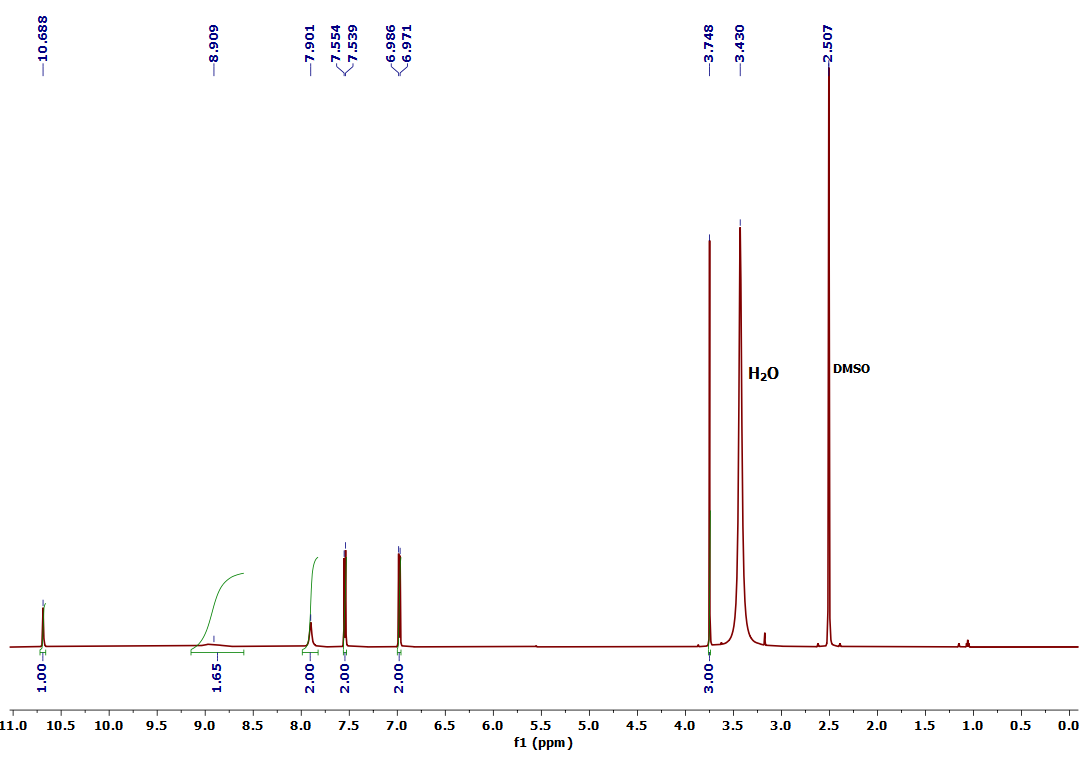


**Suppl. Fig. 2.** ^1^H NMR spectrum of title compound.


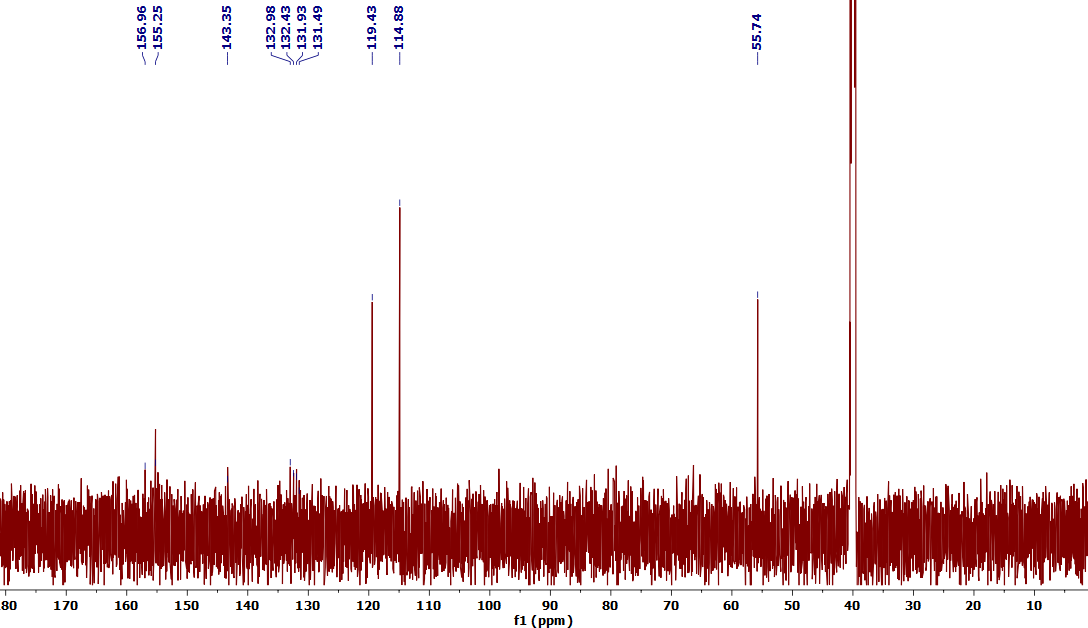


**Suppl. Fig. 3.** ^13^C NMR spectrum of title compound.





**Suppl. Fig. 4.** UV Vis. absorption spectrum of title compound.





**Suppl. Fig. 5.** Emission spectrum of title compound.
